# Potential blood biomarkers for chronic traumatic encephalopathy: The multi-omics landscape of an observational cohort

**DOI:** 10.3389/fnagi.2022.1052765

**Published:** 2022-11-07

**Authors:** Xintong Ge, Mengtian Guo, Meimei Li, Shishuang Zhang, Junlian Qiang, Luoyun Zhu, Lu Cheng, Wenzhu Li, Yan Wang, Jinwen Yu, Zhenyu Yin, Fanglian Chen, Wen Tong, Ping Lei

**Affiliations:** ^1^Haihe Laboratory of Cell Ecosystem, Department of Geriatrics, Tianjin Medical University General Hospital, Tianjin, China; ^2^Tianjin Geriatrics Institute, Tianjin, China; ^3^Department of Medical Examination, Tianjin Medical University General Hospital, Tianjin, China; ^4^Key Laboratory of Injuries, Key Laboratory of Post-trauma Neuro-repair and Regeneration in Central Nervous System, Variations and Regeneration of Nervous System, Tianjin Neurological Institute, Tianjin, China; ^5^Weightlifting, Wrestling, Judo, Boxing and Taekwondo Sports Management Center of Tianjin Sports Bureau, Tianjin, China

**Keywords:** chronic traumatic encephalopathy, traumatic encephalopathy syndrome, traumatic brain injury, biomarker, exosomes, multi-omics, transcriptomics, proteomics

## Abstract

Chronic traumatic encephalopathy (CTE) is a neurodegenerative disease associated with exposure to repetitive head impacts, which is susceptible in elderly people with declined mobility, athletes of full contact sports, military personnel and victims of domestic violence. It has been pathologically diagnosed in brain donors with a history of repetitive mild traumatic brain injury (rmTBI), but cannot be clinically diagnosed for a long time. By the continuous efforts by neuropathologists, neurologists and neuroscientists in recent 10 years, an expert consensus for the diagnostic framework of CTE was proposed in 2021 funded by the National Institute of Neurological Disorders and Stroke. The new consensus contributes to facilitating research in the field. However, it still needs to incorporate *in vivo* biomarkers to further refine and validate the clinical diagnostic criteria. From this, a single-center, observational cohort study has been being conducted by Tianjin Medical University General Hospital since 2021. As a pilot study of this clinical trial, the present research recruited 12 pairs of gender- and age-matched rmTBI patients with healthy subjects. Their blood samples were collected for exosome isolation, and multi-omics screening to explore potential diagnostic biomarkers in blood and its exosomes. The expression level of CHL1 protein, KIF2A mRNA, LIN7C mRNA, miR-297, and miR-1183 in serum and exosomes were found to be differentially expressed between groups. Besides, serum and exosomal CHL1, KIF2A, and miR-1183, as well as exosomal miR-297 were further verified as potential biomarkers for CTE by low-throughput assays. They are expected to contribute to establishing a novel set of CTE diagnostic signatures with classic neurodegenerative indicators in our future study, thereby updating the consensus diagnostic criteria for CTE by incorporating new evidence of the *in vivo* biomarkers.

## Introduction

Chronic traumatic encephalopathy (CTE) is a neurodegenerative disease associated with exposure to repetitive head impacts, typically brain concussion and subconcussive trauma (Reams et al., 2016). Elderly people with declined mobility, athletes of full contact sports, military personnel and domestic violence victims are at high risk of CTE (Thompson et al., 2006; McKee, 2020). Different from single mild head trauma which always makes full recovery within a few days, patients with CTE have a chronic disease course, characterized by progressive neurological disorders due to cumulative brain injury, named traumatic encephalopathy syndrome (TES) (Montenigro et al., 2014).

The diagnosis of CTE can be confirmed only by post-mortem neuropathologic examination demonstrating a unique pattern of perivascular hyperphosphorylated tau deposition in neurons and astrocytes at the depths of cerebral sulci (McKee et al., 2013). In order to promote clinical evaluation, diagnosis and future research for CTE, an evidence-informed, expert consensus for the diagnostic framework of CTE was developed recently by the Diagnostics, Imaging, and Genetics Network for the Objective Study and Evaluation of CTE (DIAGNOSE CTE) Research Project (Katz et al., 2021). The consensus facilitates investigations into the clinical features of TES associated with CTE pathology, and closes significant knowledge gaps, including the development of diagnostic biomarkers for CTE. However, it is still limited by the lack of objective biomarkers, resulting in a complicated and time-consuming diagnosis course based on clinical manifestations, which can only be conducted by experienced clinicians.

Although there have been several studies on fluid biomarkers for CTE pathology (Pierre et al., 2021; Bergauer et al., 2022), most of them have the disadvantage of focusing on former professional football players, but not using age-matched control groups. In addition, the research did not perform high-throughput screening, but only tested for identified indicators of other neurodegenerative diseases (e.g., neurofilament light chain protein, total tau and phosphorylated tau-181), which have been found to be lack of specificity for CTE diagnosis. Besides, many investigations collected cerebrospinal fluid samples for biomarker exploration. This is understandable, but in clinical practice, obtaining cerebrospinal fluid requires an invasive lumbar puncture, which may cause additional pain to the patient. In addition, several potential neuroimaging biomarkers with high specificity and sensitivity have been reported (Alosco et al., 2021; Bergauer et al., 2022), including Tau-PET imaging, functional MRI measures of network connectivity, MRS (magnetic resonance spectroscopy) measures of neurochemical metabolism, and structural MRI measures of volumetric and white matter injury. However, the complex technical requirements and relatively expensive medical expenditure limit the application of the above neuroimaging methods in clinical practice. Consequently, there is an urgent need to explore reliable blood biomarkers to assist clinical diagnosis for CTE.

Recently, with the development of bioinformatics, the potential value of transcriptomic and proteomics information as disease biomarkers has aroused wide attention. Exosomes are small vesicles secreted by living cells into the extracellular space that function as important carriers of information transferred among cells. They contain a large number of specific proteins and functional nucleic acids, participate in pathophysiological processes, and correlated with the occurrence and progression of various diseases. Because the membrane structure of exosomes can protect their internal ribonucleic acid (RNA) from RNase degradation, exosomal RNA is more stable and has higher concentration than free RNA in serum or plasma. Besides, in view of the maturity of the isolation and purification technique for exosomes, exosomal RNAs and proteins have been regarded as an important source of clinical biomarkers. Therefore, exploring exosomal diagnostic biomarkers for CTE would be a breakthrough point to solve the problem of early and efficient diagnosis of the disease.

In the present study, we developed a population-based matched CTE cohort, and performed multi-omics screening to explore potential diagnostic biomarkers (RNAs and proteins) in blood and its exosomes. The verified biomarkers are expected to contribute to establishing a novel set of CTE diagnostic signatures with classic neurodegenerative indicators, thereby updating the consensus diagnostic criteria for CTE by incorporating new evidence of the *in vivo* biomarkers.

## Materials and methods

### Study population and experimental design

The present research was conducted as a pilot study of the clinical trial (ClinicalTrials Identifier: NCT04928534) we hosted, named observational cohort study of blood transcriptomics and proteomics information as biomarkers of TES, which is aimed at exploring a novel set of diagnostic signatures for CTE or TES by recruiting 120 subjects with the history of rmTBI. The inclusion of human subjects was approved by the Ethics Committees of Tianjin Medical University General Hospital (Grant No. IRB2021-YX-056-01). The work began in June, 2021, and is still open to the public till now.

In this research, blood samples were collected from 12 patients with rmTBI (6 female, 6 male) who visited Cadre Physical Examination Center or Department of Neurosurgery in Tianjin Medical University General Hospital from August, 2021 to July, 2022. Blood samples from 12 gender- and age-matched healthy subjects were used as controls. Inclusion criteria for patients with rmTBI are as follows: (1) Age ≥ 18 and ≤ 80 years old with independent behavior ability. (2) Have a clear history of rmTBI. (3) The most recent head injury occurred 3 months ago. Inclusion criteria for healthy controls are as follows: (1) Age ≥ 18 and ≤ 80 years old with independent behavior ability. (2) No history of repetitive mild TBI. (3) Generally healthy with normal basic laboratory tests. Exclusion criteria include pregnant or lactating women, past history of cancer, moderate to severe traumatic brain injury, other neurological or psychiatric disorders, complications of hematological diseases, severe cardiopulmonary diseases, hepatic failure, or renal failure. More detailed information of the trial protocol is specified in the trial registration.

All patients underwent an evaluation for determining the certainty of CTE pathology according to the expert consensus, in which 1 of them were diagnosed with probable CTE (high certainty), 3 of them were diagnosed with possible CTE (medium certainty), and other 8 patients were diagnosed with suggestive of CTE (low certainty). The 4 patients with probable or possible CTE and their matched healthy individuals were selected for multi-omics high-throughput assays (RNA microarray and proteomics sequencing). Their baseline characteristics are shown in Supplementary Table 1. All subjects underwent low-throughput verification experiments [reverse transcription—polymerase chain (RT-PCR) and enzyme linked immunosorbent assay (ELISA)] for the selected candidate biomarkers, including RNAs and proteins.

### Collection of the blood and serum exosomes

Twenty milliliter blood samples were collected from all enrolled individuals in the morning after a 12-h fast and were kept in polypropylene tubes containing coagulant. They were centrifuged at 3,000 × g for 5 min at 4°C to obtain the serum (8–12 ml), and then stored at −80°C.

Six to eight milliliter serum samples were proceeded to collect exosomes using a TiO_2_ magnet protocol (Gao et al., 2019). Briefly, the serum was incubated with TiO_2_ microspheres (Q-0092910; QiYue Biology, Xi’an, China) at the concentration of 50 mg/ml for 5 min at 4°C on a thermos-shaker to allow sufficient enrichment. Exosomes were lysed from the surface of microspheres by washing three times with PBS, and were identified using electron microscopy, nanoparticle tracking analysis and surface biomarker (CD81; 1:1000, ab79559, Abcam, Cambridge, UK) detection by Western Blotting.

### Extraction of free and exosomal ribonucleic acid/protein from serum

Free RNA from the serum sample (1–1.5 ml) was extracted using TRIzol reagent (15596026; Invitrogen, Carlsbad, CA, USA) and purified with a RNeasy mini kit (74104; Qiagen, Valencia, CA, USA). Free protein from another 1–1.5 ml serum sample was extracted by a serum protein extraction kit (EX1180; Solarbio, Beijing, China). Exosomal RNA and protein from the serum sample (6–9 ml) was extracted using the Total Exosome RNA and Protein Isolation Kit (Invitrogen) following the manufacturer’s protocol.

RNA concentration of each sample was determined by NanoDrop ND-2000 Spectrophotometer (NanoDrop Technologies, Wilmington, DE, USA), and the quality was assessed with electrophoresis on 2% agarose gels. Protein concentration of each sample was quantified using spectrophotometry and the BCA (PC0020, Solarbio) method.

### Ribonucleic acid microarray

Before microarray assay, total RNA was first amplified using the GeneChip™ WT PLUS Piko Reagent Kit (902281; ThermoFisher Scientific, MA, USA). Biotinylated cDNA was then prepared from 250 ng total RNA according to the standard GeneChip™ protocol (Affymetrix, CA, USA). Following labeling, fragmented cDNA was hybridized for 16 h at 45°C on Clariom™ D Array and GeneChip™ miRNA 4.0 Array. GeneChips were then washed and stained in the GeneChip™ Fluidics Station 450. All arrays were scanned with GeneChip™ Scanner 3000 7G. The raw data was normalized by Transcriptome Analysis Console Software Version 4.0.1 with Robust Multichip Analysis algorithm using Affymetrix default analysis settings, and global scaling as a normalization method. Values presented are log2 RMA signal intensity. The threshold set for up- and down-regulated RNAs was more than 1.2-fold change with a *p*-value of less than 0.05. Hierarchical clustering was performed based on differentially expressed lncRNAs (DElncRNAs), circRNAs (DEcircRNAs), miRNAs (DEmiRNAs), and mRNAs (DEmRNAs) using R package Pheatmap Version 1.0.12 (OE biotech, Shanghai, China).

### Proteomic sequencing

The protein sample was digested, prepared for transition library construction, and scanned by mass spectrometry using the data-independent acquisition (DIA) mode by Orbitrap mass spectrometer (Q Exactive HF-X, Thermo Fisher Scientific). The MS data of the single-shot subject samples were used to generate a hybrid library for protein identification and quantitation using Spectronaut software Version 15.7 (Biognosys AG, Switzerland). All searches were performed against the human UniProt reference proteome of canonical and isoform sequences with 20,373 entries downloaded in March 2022. Searches used carbamidomethylation as fixed modification, and acetylation of the protein N-terminus as well as oxidation of methionines as variable modifications. The trypsin/P proteolytic cleavage rule was applied, permitting a maximum of two miscleavages and a peptide length of 7–52 amino acids. Protein intensities were normalized using the Local Normalization algorithm based on a local regression model (Callister et al., 2006). Spectral library generation stipulated a minimum of three fragments per peptide, and maximally, the six best fragments were included. A *Q*-value of 1% for the precursor and protein were used, and protein quantities were reported only in samples that passed the filter. The significantly differentially expressed proteins (DEPs) were screened out if their absolute ratio of fold change > 1.20 with *Q*-value < 0.05 (OE biotech).

### Gene ontology and Kyoto Encyclopedia of Genes and Genomes enrichment analyses for co-DEmRNAs and target genes of co-DEmiRNAs

Genes that were simultaneously differentially expressed in serum and its exosomes with the same trend (both up-regulated or down-regulated) were obtained for subsequent analysis. They were named as co-DEmRNAs, co-DEmiRNAs, co-DElncRNAs, and co-DEcircRNAs.

The Gene Ontology (GO) is a structured and controlled vocabulary of terms, which are subdivided into 3 non-overlapping ontologies, Molecular Function, Biological Process and Cellular Component. GO enrichment analysis was performed to analyze the primary function of a list of genes using the GO database.^1^ Kyoto Encyclopedia of Genes and Genomes (KEGG) is a knowledge base for systematic analysis of gene functions, linking the genomic information with higher order functional information. The pathway enrichment analysis was used to find out the significant pathway of a list of genes using the KEGG database.^2^ According to the obtained miRNA-mRNA interaction, GO and KEGG enrichment analyses for co-DEmRNAs and target genes of co-DEmiRNAs were carried out using the enrichment analysis tool of Metascape.^3^

### Prediction of miRNA-mRNA, lncRNA-miRNA, and circRNA-miRNA regulation relationship

The interaction of miRNA-mRNA was predicted using the miRWalk 3.0,^4^ an integrated public miRNA-target prediction database that includes the data from PITA, miRmap, miRanda, PicTar, and TargetScan. The predicted miRNA-mRNA pairs were then integrated with the data of co-DEmiRNAs and co-DEmRNAs to obtain their regulatory relationships.

The lncRNA-miRNA and circRNA-miRNA interaction prediction was performed using the ENCORI,^5^ a website identified 1.5 million of RNA-RNA interactions through analyzing thousands of CLIP-seq and various high-throughput sequencing data. The screening criteria were number of supporting experiments low stringency R1, and the predicted lncRNA-miRNA and circRNA-miRNA pairs were integrated with the data of co-DElncRNAs and co-DEcircRNAs to obtain their regulatory relationships.

### Co-expression analysis of co-DElncRNAs, co-DEcircRNAs, and co-DEmRNAs

Using the matrix data of co-DElncRNAs, co-DEcircRNAs, and co-DEmRNAs, the Pearson correlation coefficients was calculated for each lncRNA-mRNA and circRNA-mRNA pair. A correlated test was then performed to find significant pairs with the threshold of | r| > 0.9 and *p* < 0.05.

### Construction of competing endogenous RNAs network

The ceRNA network was constructed to discover the ceRNA mechanism based on co-DERNAs, including co-DElncRNAs, co-DEcircRNAs, co-DEmiRNAs, and co-DEmRNAs. Briefly, the Pearson Correlation Coefficient between matched miRNA-mRNA, lncRNA-miRNA, circRNA-miRNA, lncRNA-Mrna, and circRNA-mRNA was computed using R function cor.test (Hmisc and corplot) based on their expression data. The significant correlation pair was defined with a correlation value of more than 0.99. For a given lncRNA-mRNA or circRNA-mRNA pair, both of them were targeted by a common miRNA, and co-expressed negatively with it. Such a lncRNA-miRNA-mRNA or circRNA-miRNA-mRNA pair was identified as competing ceRNA triplets. The two complex networks were finally integrated, and the visualization of the ceRNA network was built by software Cytoscape Version 3.6.0.

### Construction of protein-protein interaction network

Based on the interactions of proteins in the KEGG database, global signal transduction network was generated to demonstrate the interaction among the DEPs, in order to select the key proteins with powerful ability to modulate others in the network. The visualization of PPI network was built by software Cytoscape Version 3.6.0. Briefly, to get the most closely related interaction pairs, the PPI score was set to 0.99 (high confidence). The CytoHubba plug-in Version 0.1 was used to analyze the network topology properties of the nodes, and obtain the significant ones by score ranking. The MCODE plug-in Version 2.0.2 was then used to analyze the most important clustering modules in the PPI network with a score threshold of more than 10.

### Multi-omics analysis strategy

The results of high-throughput assays on blood samples from the 8 subjects include 4 datasets: serum free RNAs, serum free proteins, exosomal RNAs and exosomal proteins. Multi-omics analysis was performed following the steps as below: (1) Obtained serum and exosomal differentially expressed indicators on both mRNA and protein level. They were analyzed as the first part of candidate CTE biomarkers (KIF2A, FCER1G, GNAZ, RAP1B, and RSU1). (2) Obtained the intersection of serum free DEPs and exosomal DEPs. The indicators in the two intersections were analyzed as the second part of candidate CTE biomarkers (CHL1 and CFB). (3) Screened out hub genes with the highest connectivity in the ceRNA network as the third part of candidate CTE biomarkers (LIN7C, hsa-miR-297, hsa-miR-1183, NON-HSAT081066.2, AND NON-HSAT205595.1). (4) Screened out hub genes with the highest degree of link in the PPI network as the fourth part of candidate CTE biomarkers (APOA1, CFB, C1QA, C1QB, C1QC, and CD9). (5) Manually search the function of above candidate biomarkers using the GeneCards database^6^ and the RNAdisease database,^7^ and selected the genes (CHL1, KIF2A, LIN7C, hsa-miR-297, and hsa-miR-1183) that associated with neuropathology to ensure the diagnostic specificity. Their expression levels were further determined by low-throughput assays for blood samples from all 24 subjects in the cohort.

### Enzyme linked immunosorbent assay

Low-throughput verification for potential protein biomarkers of CTE were performed using ELISA. The CHL1 concentration was determined by the ELISA kit (ABIN5510733; antibodies-online, Aachen, Germany) following the manufacturer’s instructions.

### Reverse transcription—polymerase chain reaction

Low-throughput verification for potential RNA biomarkers of CTE were performed using RT-PCR. Briefly, reverse transcription and PCR were performed using the Hairpin-it miRNA/mRNA RT-PCR Quantitation kit (GenePharma, Shanghai, China) with corresponding primers. GAPDH and U6 were used as the internal control for mRNA and miRNA quantification, respectively. All PCR reactions were performed using standard PCR conditions. The Ct value was detected by CFX Connect™ RT-PCR system (Bio-Rad, Hercules, CA USA), and the data were analyzed by means of 2^–ΔΔ*Ct*^ method on each group. The primers used were as follows: GAPDH, GCCAAGGCTGTGGGCAAGGT (forward) and TCTCCAGGCGGCACGTCAGA (reverse); U6, CTCGCTT CGGCAGCACA (forward) and AACGCTTCACGAATTT GCGT (reverse); KIF2A, ATTTTCTCTCATTGACCTGGCTG (forward) and ACTCCTTGAGTGCTAAAAGGC (reverse); LIN7C, TGCCGCCACAGAAACTTCAG (forward) and TGATGTCCACCGTCTCATAAACA (reverse); hsa-miR-297, GCAGATGTATGTGTGCATGTG (forward) and AGGTCCAGTTTTTTTTTTTTTTTCAT (reverse); hsa-miR-1183, CACTGTAGGTGATGGTGAGA (forward) and AGTTTTTTTTTTTTTTTGCCCACT (reverse).

### Statistical analysis

The continuous variables were expressed as mean ± standard deviation, and compared using the Student’s *t*-test or Wilcoxon rank sum test as appropriate. Categorical data were expressed as number (percentage) and compared using the chi-square test. Statistical analyses for GO and KEGG enrichment analysis were performed using Fisher’s exact test. Pearson’s correlation test was utilized to calculate the correlation coefficients between hub genes in the PPI network. All statistical analyses were performed using SPSS Statistics Version 27.0 (IBM, Armonk, NY, USA). A two-tailed *p*-value of less than 0.05 was considered to be statistically significant.

## Results

### Baseline characteristics of enrolled subjects

Twelve pairs of gender- and age-matched rmTBI patients with healthy subjects were enrolled in this research, and their demographic characteristics are presented in Table 1. First, no difference of age and gender was observed between groups. Second, we evaluated the degree of CTE diagnosis for the enrolled subjects using a grading method as follows: (1) Probable CTE. (2) Possible CTE. (3) Suggestive of CTE or only with rmTBI history. (4) Healthy control. Therefore, a lower score represents a higher possibility of clinical diagnosis, as patients with rmTBI history had a lower score than healthy subjects. Third, cognitive and neurobehavioral tests, including Mini-Mental State Examination (MMSE) (Folstein et al., 1975), Montreal Cognitive Assessment (MoCA) (Nasreddine et al., 2005) and Rivermead Post-Concussion Symptoms Questionnaire (RPQ) (King et al., 1995) as the consensus suggested. We found that rmTBI patients performed poorly on the tests compared with the healthy subjects, which suggested greater cognitive impairment and neurobehavioral dysregulation.

**TABLE 1:** Baseline characteristics of enrolled patients with rmTBI and control subjects.

Variables	rmTBI (*n* = 12)	Healthy (*n* = 12)	*P*-value
Age, Years	50.00 ± 3.11	49.08 ± 2.71	0.826
Male, n (%)	6 (50.0)	6 (50.0)	1.000
CTE degree	2.58 ± 0.19	4.00 ± 0.00	< 0.001***
**Cognitive and neurobehavioral tests**			
MoCA	25.08 ± 0.72	29.33 ± 0.36	< 0.001***
MMSE	27.66 ± 0.82	29.83 ± 0.17	0.020*
RPQ-3	1.75 ± 0.46	0.08 ± 0.08	< 0.001***
RPQ-16	7.00 ± 1.75	1.17 ± 0.48	0.001**

The data were expressed as mean ± SD or *n* (%). *P < 0.05, **P < 0.01, ***P < 0.001. CTE, chronic traumatic encephalopathy; MMSE, Mini-Mental State Examination; MoCA, Montreal Cognitive Assessment; RPQ, Rivermead Post-Concussion Symptoms Questionnaire.

### Acquisition of DERNAs and differentially expressed proteins in serum and exosomes

**FIGURE 1:**
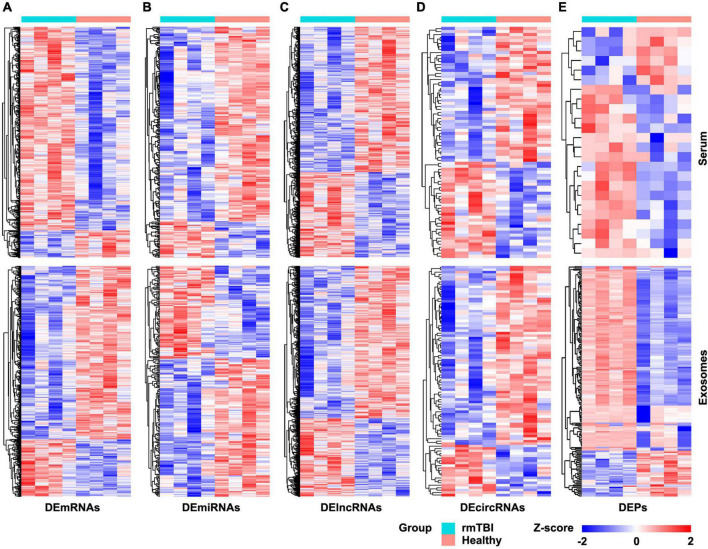
Heatmap of DERNAs and DEPs in serum and exosomes between patients with rmTBI and healthy subjects. **(A)** DEmRNAs in serum and exosomes. **(B)** DEmiRNAs in serum and exosomes. **(C)** DElncRNAs in serum and exosomes. **(D)** DEcircRNAs in serum and exosomes. **(E)** DEPs in serum and exosomes. Up-regulated genes are shown in red, and down-regulated genes are shown in blue. DE, differentially expressed; DEPs, differentially expressed proteins.

The results of RNA microarray and proteomic sequencing for 4 pairs of gender- and age-matched CTE patients with healthy subjects were comprehensively analyzed, and the original data were uploaded as Supplementary Data Sheets. As shown in Figure 1, the distribution of DERNAs and DEPs were nearly identical after normalization within a group. According to the screening criterion, 1,189 DEmRNAs (1,049 up-regulated and 140 down-regulated), 249 DEmiRNAs (39 up-regulated and 210 down-regulated), 4,235 DElncRNAs (1,412 up-regulated and 2,823 down-regulated), 77 DEcircRNAs (32 up-regulated and 45 down-regulated), and 24 DEPs (17 up-regulated and 7 down-regulated) were found in serum. In addition, 1,359 DEmRNAs (335 up-regulated and 1,024 down-regulated), 251 DEmiRNAs (101 up-regulated and 140 down-regulated), 3,759 DElncRNAs (1,189 up-regulated and 2,570 down-regulated), 94 DEcircRNAs (21 up-regulated and 73 down-regulated), and 190 DEPs (154 up-regulated and 36 down-regulated) were identified in serum exosomes.

### Gene ontology and Kyoto Encyclopedia of Genes and Genomes enrichment analyses for co-DEmRNAs and target genes of co-DEmiRNAs

**FIGURE 2:**
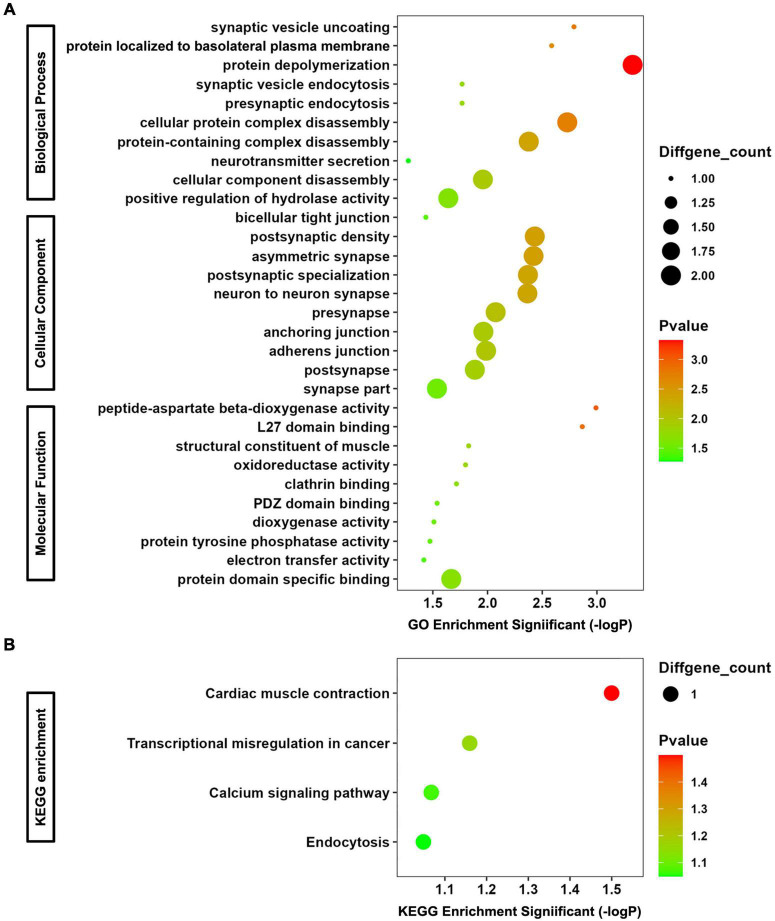
GO and KEGG Enrichment Analyses for co-DEmRNAs and target genes of co-DEmiRNAs. **(A)** Bubble chart of GO analysis. **(B)** Bubble chart of KEGG analysis.

The co-DEmRNAs and co-DEmiRNAs were defined as mRNAs and miRNAs that were both up-regulated or down-regulated in serum and its exosomes of the 4 CTE patients, compared with healthy subjects. A functional enrichment analysis was performed for co-DEmRNAs and target genes of co-DEmiRNAs, and the top 10 GO and all KEGG pathway terms are shown in Figure 2.

In terms of biological progress of GO analysis, the genes were significantly enriched in synaptic vesicle uncoating, protein localized to basolateral plasma membrane, protein depolymerization, synaptic vesicle endocytosis, presynaptic endocytosis, cellular protein complex disassembly, protein-containing complex disassembly, neurotransmitter secretion, cellular component disassembly, and positive regulation of hydrolase activity, most of which are associated with synaptic function. Regarding cellular component of GO analysis, the enriched terms included bicellular tight junction, postsynaptic density, asymmetric synapse, postsynaptic specialization, neuron to neuron synapse, presynapse, anchoring junction, adherens junction, postsynapse, and synapse part. The function of them are related to tight junction (important component of blood-brain barrier) and neurotransmitter. As to molecular function of GO analysis, peptide-aspartate beta-dioxygenase activity, L27 domain binding, structural constituent of muscle, oxidoreductase activity, clathrin binding, PDZ domain binding, dioxygenase activity, protein tyrosine phosphatase activity, electron transfer activity, and protein domain specific binding were enriched. For KEGG analysis, the most significantly enriched pathways were cardiac muscle contraction, transcriptional misregulation in cancer, calcium signaling pathway and endocytosis. Consequently, changes in the expression levels of co-DEmRNAs and target genes of co-DEmiRNAs in serum and exosomes may reflect altered function on synapse-mediated neurotransmitter transmission and blood-brain barrier permeability after rmTBI.

### Competing endogenous RNA network of co-DERNAs

**FIGURE 3:**
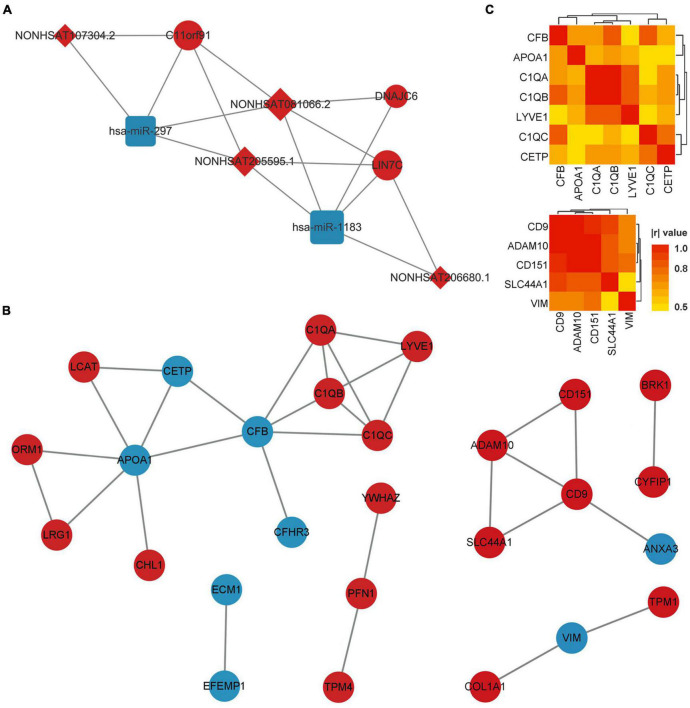
ceRNA network of co-DERNAs and PPI network of DEPs in serum and exosomes. **(A)** ceRNA network of co-DERNAs. A circle represents a mRNA, a square represents a miRNA, a rhombus represents a lncRNA, and relationships between them are represented by edges. **(B)** PPI network of DEPs in serum and exosomes. Nodes represent the proteins, and edges represent relationships. Up-regulated genes and proteins are shown in red, and down-regulated genes and proteins are shown in blue. **(C)** Heatmap of correlations among the hub genes in the PPI network of DEPs in serum and exosomes. PPI, protein-protein interaction; DEPs, differentially expressed proteins; DERNAs, differentially expressed RNAs.

Using the matrix data of co-DERNAs and online tools to predict their regulation relationship, 4 DElncRNAs were found to target 2 DEmiRNAs, which are able to bind 3 DEmRNAs. A ceRNA network was thus constructed according to the lncRNA-miRNA-mRNA triplets (Figure 3A). Then, the hub genes with the highest connectivity in the network were acquired as candidate CTE biomarkers (LIN7C, hsa-miR-297, hsa-miR-1183, NON-HSAT081066.2, AND NON-HSAT205595.1) for further analysis.

### Protein-protein interaction network and correlations of differentially expressed proteins in serum and exosomes

The PPI network of DEPs in serum and exosomes were constructed individually based on their interaction relationship in the KEGG database. As shown in Figure 3B, we found 17 hub genes in the PPI network of serum DEPs, which included APOA1, C1QA, C1QB, C1QC, CETP, CFB, CFHR3, CHL1, ECM1, EFEMP1, LCAT, LRG1, LYVE1, ORM1, PFN1, TPM4, and YWHAZ. In addition, 10 hub genes were screened out from the PPI network of exosomes DEPs. They were ADAM10, ANXA3, BRK1, CD151, CD9, COL1A1, CYFIP1, SLC44A1, TPM1, and VIM.

Hub genes with more than 3 links in the PPI network of DEPs in serum, and those with more than 2 links in the PPI network of DEPs in exosomes were selected for the Pearson correlation analysis (Figure 3C). We found that no DEP showed a | r| value of more than 0.8 (or *p*-values of less than 0.01) with all the other DEPs. In addition, GO and KEGG Enrichment Analyses were performed, which indicated that the genes were enriched in synapse pruning, cell junction disassembly, integrin binding complement activation, immune effector process, regulation of immune system process, and complement/coagulation cascades.

### Potential diagnostic biomarkers for chronic traumatic encephalopathy confirmed by low-throughput assays

**FIGURE 4:**
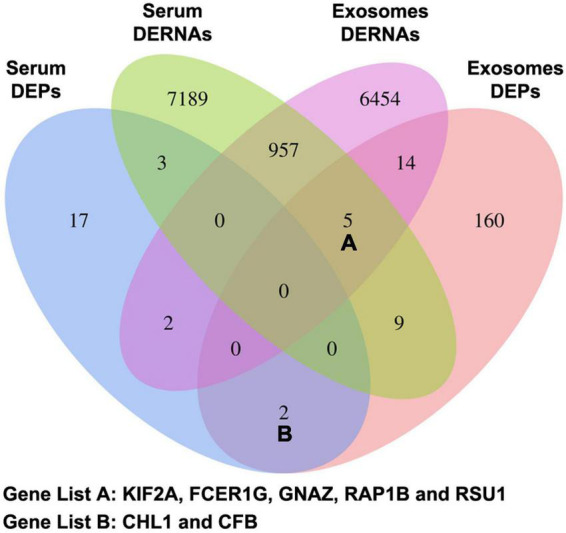
Venn map of DERNAs and DEPs in serum and exosomes between patients with rmTBI and healthy subjects. DERNAs, differentially expressed RNAs; DEPs, differentially expressed proteins.

**FIGURE 5:**
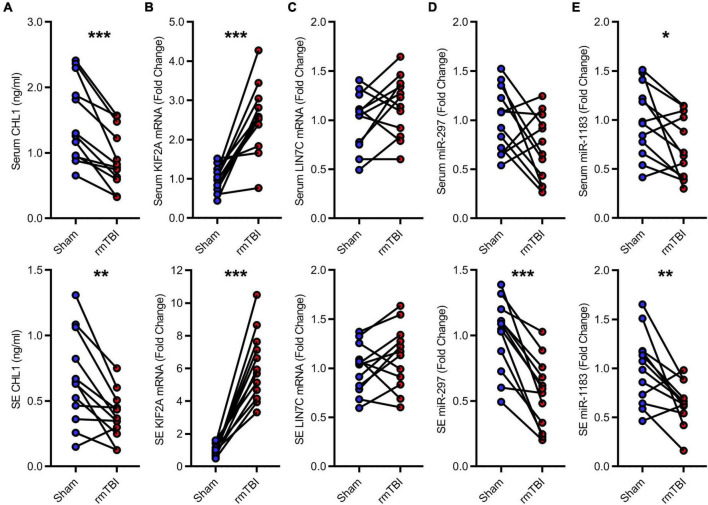
Low-throughput assays for candidate diagnostic biomarkers for CTE. **(A)** Quantitative data of ELISA for serum and exosomal CHL1 level in indicated groups. **(B–E)** Quantitative data of RT-PCR for serum and exosomal KIF2A, LIN7C, hsa-miR-297, hsa-miR-1183. *n* = 12 per group. Mean ± SD. *P < 0.05, **P < 0.01, ***P < 0.001. Statistical analyses were performed using two-tailed paired Student’s *t*-test. SE, serum exosomes.

The candidate CTE biomarkers (CHL1 protein, KIF2A mRNA, LIN7C mRNA, hsa-miR-297, and hsa-miR-1183) were screened out through multi-omics analysis using Venn map (Figure 4) and functional investigation by online database. Their expression levels were further determined by ELISA and RT-PCR for blood samples from all 24 subjects in the cohort. We found that serum and exosomal CHL1 protein level was decreased in rmTBI patients compared with the healthy controls, which are consistent with the results of proteomic sequencing (Figure 5A). Besides, the mRNA level of KIF2A in serum and exosomes were both up-regulated in patients with rmTBI (Figure 5B). However, no difference was observed on serum and exosomal LIN7C mRNA level, as well as serum miR-297 level between patients with rmTBI and healthy subjects, suggesting that they are not suitable biomarkers for CTE (Figures 5C,D). In addition, exosomal miR-297, serum miR-1183, and exosomal miR-1183 levels were all down-regulated in rmTBI patients (Figures 5D,E). These findings indicated that serum and exosomal CHL1, KIF2A and miR-1183, as well as exosomal miR-297 are the potential diagnostic signatures for CTE.

## Discussion

Patients with CTE are characterized by progressive neurological disorders. Their clinical symptoms mainly include cognitive decline, behavioral change, emotional dysregulation and motor disturbance (Reams et al., 2016). The cognitive decline typically affects more than one domain of neurological deficits including executive, visuospatial, memory, and language. Patients with mainly behavioral change may exhibit violence, poor impulse control, socially inappropriate behavior, avolition, apathy, change in personality, and comorbid substance abuse. The emotional dysregulation includes depression, anxiety, agitation, aggression, paranoid ideation, deterioration of interpersonal relationships, and suicidality, while the motor disturbance contains bradykinesia, tremor, rigidity, gait instability, dysarthria, dysphagia, ataxia, and gaze disturbance. Consequently, the clinical symptoms of CTE could be very difficult to identify, which leads to a complicated and time-consuming diagnosis course in accordance with the current expert consensus.

In order to explore novel blood signatures to improve the diagnostic framework of CTE, we started the observational cohort study of blood transcriptomics and proteomics information as biomarkers of TES in 2021. In the present research, we collected blood samples from the first 12 pairs of rmTBI patients with healthy volunteers that had been recruited for the cohort. By high-throughput multi-omics screening, we found that co-DEmRNAs, target genes of co-DEmiRNAs and hub genes of the PPI network in serum and exosomes were significantly enriched in synapse-mediated neurotransmitter transmission and blood-brain barrier permeability. It is worth noting that chronic synaptic adaptation and reorganization, as well as blood-brain barrier breakdown and repair are indeed important neuropathological changes in CTE, and associated with the cognitive dysfunction (Sweeney et al., 2018; Kim et al., 2021; Sloley et al., 2021). On this basis, serum and exosomal CHL1, KIF2A, and miR-1183, as well as exosomal miR-297 were further identified to be potential diagnostic biomarkers for CTE using low-throughput verification experiments.

CHL1 protein is a member of the L1 gene family of neural cell adhesion molecules. It is a neural recognition molecule that involves in L1CAM interactions, nervous system development and synaptic plasticity. Specifically, it could promote neurite outgrowth of hippocampal neurons, suppress neuronal cell death, and regulate cell migration in nerve regeneration and cortical development. CHL1 also plays a role in positioning of pyramidal neurons, regulating the number of interneurons and the efficacy of GABAergic synapses, and potentiating integrin-dependent cell migration toward extracellular matrix proteins (Schmid and Maness, 2008; Irintchev and Schachner, 2012). These functions are consistent with our findings on GO and KEGG enrichment analysis, in which synapse, tight junction and related biological progress were significantly enriched. Therefore, the observed decreased CHL1 protein level suggested the neural and synaptic damage following rmTBI. KIF2A, named Kinesin Family Member 2A, encodes protein that is required for normal spindle activity during mitosis and brain development. Its dysfunction is associated with cortical dysplasia and brain malformations. KIF2A may also regulate synaptic transmission and axonal transport coupled with neuroinflammation, as well as microtubule dynamics during axonal growth (Chung et al., 2009; Niwa, 2015). As neural inflammation is the most crucial neuropathology that leads to neural death, neurodegeneration and subsequent cognitive impairment (Morganti-Kossmann et al., 2019), it’s up-regulation in serum and exosomes observed in rmTBI patients may be a manifestation of the neuroprotective compensatory mechanism. miR-297 and miR-1183 are potential biomarkers for a series of neurological diseases, including ischemic stroke, intracerebral hemorrhage, intracranial aneurysm, Moyamoya disease, Parkinson’s disease, and glioma manifested by the RNAdisease database. From this, it could be inferred that their decrease following rmTBI may be an indicator of both acute and chronic brain injury.

The verified biomarkers are expected to contribute to CTE diagnosis. However, several limitations, especially the small cohort size should be taken into account when interpreting the results. First, according to the provisional levels of certainty for CTE pathology proposed by the expert consensus, the patients we recruited were classified as 1 probable CTE, 3 possible CTE and 8 suggested of CTE or only with rmTBI history. Due to the low incidence of the disease, we failed to follow the opinions of expert consensus, in which cohorts of patients with probable CTE as primary subjects are recommended for the biomarker study. It may lead to a decrease in the specificity of the biomarkers we found. Second, the small sample size for high-throughput sequencing and low-throughput verification assays may result in the screened differentially expressed RNAs and proteins cannot well reveal characteristics of the whole CTE population due to individual differences. Therefore, we did not depict Receiver-Operating Characteristic curve to show the outcome prediction performance of each potential biomarker, or compare with that of classic neurodegenerative biomarkers, in order to avoid their misuse in future research and clinical diagnosis.

## Conclusion

In conclusion, this research is a pilot study of our ongoing cohort study (ClinicalTrials.gov Identifier: NCT04928534). It provides effective methodologies for managing subsequent large-scale cohort study and analyzing the multi-omics big data. Although the potential biomarkers for CTE we found are not recommended to be directly applied to clinical use, they contribute to further studies on establishing a novel set of CTE diagnostic signatures with classic neurodegenerative indicators. As specified in the trial registration, our research team will continue to develop a CTE cohort including 120 strictly matched rmTBI patients and healthy subjects, aiming to update the consensus diagnostic criteria by incorporating solid evidence of the *in vivo* biomarkers.

## Data availability statement

The RNA microarray and proteomic sequencing datasets for this study can be found in the ArrayExpress database (Accession numbers: E-MATB-12328, E-MATB-12329, E-MATB-12330, and E-MATB-12331) and the ProteomeXchange database (Project ID: PXD037637). The processed matrix data of high-throughput assays can be found in the Supplementary material.

## Ethics statement

The studies involving human participants were reviewed and approved by the Ethics Committees of Tianjin Medical University General Hospital (Grant No. IRB2021-YX-056-01). The patients/participants provided their written informed consent to participate in this study.

## Author contributions

XG and PL were responsible to conception and design of the study. XG developed the method, performed data visualization, statistical analysis, and wrote the manuscript. ML, MG, and LZ conducted laboratory examinations. SZ, JQ, LC, WL, YW, JY, ZY, and WT contributed to cohort recruitment. FC provided methodological supports. PL reviewed the manuscript. All authors contributed to manuscript revision, read, and approved the submitted version.

## Footnotes

1
http://geneontology.org/

2
https://www.genome.jp/kegg/

3
https://metascape.org

4
http://mirwalk.umm.uni-heidelberg.de/

5
https://starbase.sysu.edu.cn/index.php

6
https://www.genecards.org/

7
http://www.rnadisease.org/

## Abbreviations

CTE: chronic traumatic encephalopathy
ELISA: Enzyme Linked Immunosorbent Assay
GO: Gene Ontology
KEGG: Kyoto Encyclopedia of Genes and Genomes
PPI: protein-protein Interaction
RT-PCR: Reverse Transcription—Polymerase Chain Reaction
TES: traumatic encephalopathy syndrome.
