# Supplementary material for: Potential blood biomarkers for chronic traumatic encephalopathy: The multi-omics landscape of an observational cohort
Source: Front Aging Neurosci. 2022 Nov 7;14:1052765. doi: 10.3389/fnagi.2022.1052765 (PMC9676976; doi:10.3389/fnagi.2022.1052765)
Supplement: Supplementary file 13 [file Table_1.DOCX]

**Table S1.** Baseline characteristics of 4 paired patients with traumatic encephalopathy syndrome and control subjects.

| **CTE Certainty** | Probable | Healthy | Possible | Healthy |
| --- | --- | --- | --- | --- |
| **Past history** | Headache (1); Dizziness (1); Forgetfulness (3); Nausea (1); Fatigue (1); Depressed (1); Poor concentration (1); Longer to think (1); Restlessness (1) | - | Dizziness (1); Forgetfulness (2); Sleep disturbance (4); Fatigue (2); Irritable (3); Depressed (2); Frustrated (2); Poor concentration (1); Longer to think (1); Blurred vision (1) | - |
| **RPQ-16** | 11 | 0 | 19 | 0 |
| **RPQ-3** | 5 | 0 | 3 | 0 |
| **MMSE** | 26 | 30 | 25 | 30 |
| **MoCA** | 24 | 30 | 22 | 30 |
| **Time since last injury** | 4 years | - | 1 year | - |
| **mTBI number** | FCS  13 years | 0 | FCS  10 years | 0 |
| **Gender** | Female | Female | Female | Female |
| **Age** | 41 | 40 | 44 | 43 |
| **Subjects Number** | **rmTBI #2** | **Healthy #2** | **rmTBI #3** | **Healthy #3** |

| **CTE Certainty** | Possible | Healthy | Possible | Healthy |
| --- | --- | --- | --- | --- |
| **Past history** | Headache (1); Forgetfulness (2); Sleep disturbance (2); Fatigue (1); Irritable (2); Poor concentration (2); Longer to think (2) | Forgetfulness (1); Sleep disturbance (1); Fatigue (1); Irritable (1); Longer to think (1) | Headache (1); Dizziness (2); Forgetfulness (1); Nausea (1); Sleep disorder (4); Fatigue (1); Irritable (1) Depressed (1); Frustrated (1); Poor concentration (1); Longer to think (1); Restlessness (1) | Sleep disorder (1); Fatigue (1); Irritable (1) |
| **RPQ-16** | 12 | 5 | 16 | 3 |
| **RPQ-3** | 3 | 1 | 4 | 0 |
| **MMSE** | 24 | 28 | 22 | 30 |
| **MoCA** | 22 | 26 | 20 | 28 |
| **Time since last injury** | 3 year | - | 1 year | - |
| **mTBI number** | 4 | 0 | 4 | 0 |
| **Gender** | Female | Female | Male | Male |
| **Age** | 71 | 68 | 62 | 59 |
| **Subjects Number** | **rmTBI #7** | **Healthy #7** | **rmTBI #8** | **Healthy #8** |

(Continued Table S1)

Degree of symptoms: (1) no more of a problem, (2) a mild problem, (3) a moderate problem, (4) a severe problem. Abbreviations: CTE, chronic traumatic encephalopathy; FCS, full contact sports; MoCA, Montreal Cognitive Assessment; MMSE, Mini-Mental State Examination; RPQ, Rivermead Post-Concussion Symptoms Questionnaire.
